# Predictors and temporal trend of flu vaccination in auto-immune rheumatic diseases in the UK: a nationwide prospective cohort study

**DOI:** 10.1093/rheumatology/key156

**Published:** 2018-06-12

**Authors:** Georgina Nakafero, Matthew J Grainge, Puja R Myles, Christian D Mallen, Weiya Zhang, Michael Doherty, Jonathan S Nguyen-Van-Tam, Abhishek Abhishek

**Affiliations:** 1 Academic Rheumatology, University of Nottingham, Nottingham, UK; 2 Epidemiology and Public Health, University of Nottingham, Nottingham, UK; 3 Primary Care Sciences, Keele University, Keele, UK

**Keywords:** rheumatoid arthritis, influenza, vaccination, disease modifying anti-rheumatic drugs

## Abstract

**Objectives:**

To examine temporal trend in uptake of seasonal influenza vaccine (SIV) in the UK and explore disease and demographic factors associated with vaccination.

**Methods:**

From the Clinical Practice Research Datalink, 32 751 people with auto-immune rheumatic diseases prescribed DMARDs between 2006 and 2016 were identified. The proportion vaccinated between 1 September of one year and 31 March of the next year was calculated and stratified by age, other indications for vaccination, auto-immune rheumatic diseases type and number of DMARDs prescribed. Stata and Joinpoint regression programs were used.

**Results:**

SIV uptake was high in those aged ⩾65 years (82.3 and 80.7% in 2006–07 and 2015–16, respectively). It was significantly lower in other age groups, but improved over time with 51.9 and 61.9% in the 45–64 year age group, and 32.3 and 50.1% in the <45 year age group being vaccinated in 2006–07 and 2015–16, respectively. While 64.9% of the vaccinations in those ⩾65 years old occurred by 3 November, in time to mount a protective immune response before the influenza activity becomes substantial in the UK, only 38.9% in the 45–64 year and 26.2% in the <45 year age group without any other reason for vaccination received SIV by this date. Women, those with additional indications for vaccination, on multiple DMARDs and with SLE were more likely to be vaccinated.

**Conclusion:**

SIV uptake is low in the under 65s, and the majority of them are not vaccinated in time. Additional effort is required to promote timely uptake of SIV in this population.

**Rheumatology key messages**

- Uptake of seasonal influenza vaccine is low in the absence of additional indications for vaccination.
- Most vaccinations do not occur in time before the flu virus begins to circulate.
- Young age seems to be an important barrier to seasonal influenza vaccination.

## Introduction

Autoimmune rheumatic diseases (AIRDs) such as RA and SLE associate with increased risk of influenza and its complications [1–4]. This may be compounded by the use of potent DMARDs and biologic agents [5–8]. The EULAR and ACR recommend that people with AIRDs receive seasonal influenza vaccine (SIV) before commencing a DMARD, and annually thereafter [9, 10]. In the UK, SIV is recommended for people >65 years, the immunosuppressed and those at risk of influenza or its complications [11], while universal vaccination is recommended for adults in the USA [12].

The uptake of SIV in people with RA, the commonest AIRD, is suboptimal. For instance, only 23.5% of people with RA in the multinational COMObidities of Rheumatoid Arthritis (COMORA) cohort received SIV with substantial global variation (range 1–66.2%) [13]. Higher self-reported vaccination rates (range 37–77%) were reported in small (*n* = 71–155) hospital-based surveys from the UK [14–19]. Similar small (*n* = 100–137) single-centre hospital-based studies from other European hospitals reported uptakes of 20–48% [20–22]. A previous study using data from the Clinical Practice Research Datalink (CPRD) reported that 80% of incident RA cases on DMARDs receive at least one SIV over a mean 5-year period, with 21–31% of the under 65 years and 55–76% of those 65 years in age or older receiving all expected vaccinations [23] This and other small single-centre studies mostly include people with RA, and a large study examining the uptake of SIV in a range of AIRDs has not been performed. Similarly, it is not known whether the uptake of SIV is improving and whether people with AIRDs are vaccinated in time, that is, at least 2 weeks before commencement of influenza activity to allow time for seroconversion.

The overall aim of this study was to determine the uptake of SIV in people with AIRDs treated with DMARDs. The objectives of this study were to examine (i) the temporal trend in SIV uptake between 2006–07 and 2015–16 influenza seasons, (ii) the proportion vaccinated in time before the seasonal flu virus circulates in the community, (iii) factors associated with receiving SIV and (iv) regional variation in vaccination in people with AIRDs treated with DMARDs.

## Methods

### Data source

CPRD is a longitudinal database of the UK’s general practice (GP) medical records incepted in 1987. In July 2013, 684 GPs were contributing data to CPRD for over 11 million anonymized patients [24]. People registered in the CPRD are representative of the UK general population in terms of age, sex and ethnicity. CPRD includes information on demographics, lifestyle factors, diagnoses, medications, results of investigations and examinations, referral to hospitals, and prescribed medications. Diagnoses in CPRD are recorded using Read codes, a coded dictionary of clinical terms. The data undergo thorough quality checks and are of a reliable research standard with a high validity of recorded diagnoses, including a median proportion of cases with a confirmed diagnosis of 89% for 183 different conditions including chronic auto-immune diseases [25]. Validation studies also confirm high levels of completeness of clinical, diagnostic and prescription data [25].

This study was approved by the Independent Scientific Advisory Committee of the Medicines and Healthcare Regulatory Authority (reference number: 16_288R).

### Study population

Participants having one or more Read codes for RA, SLE or spondyloarthropathy (SpA, defined as PsA, ReA, IBD-associated arthritis or AS) and at least one prescription of MTX, LEF, SSZ, AZA, ciclosporin, MMF or tacrolimus between 1 April 2006 and 31 March 2016 were included.

### Identification of participants

Previously published Read code lists were used to identify people with RA [26, 27]. Read code lists to identify participants with SLE, ReA, IBD-associated arthritis, AS or PsA were developed by G.N. (a post-doctoral research fellow) A.A. (a rheumatologist) and C.D.M. (a general practitioner). Similarly, product code lists were developed to identify prescriptions of MTX, LEF, SSZ, AZA, ciclosporin, MMF and tacrolimus.

Entry into the study was considered as the latest of DMARD prescription date, current registration date, or 1 April 2006. Study exit was the earliest of transfer out date, date of death, date of last data collection from the participants’ GP surgery or 31 March 2016.

### Cohort building

The study period 1 April 2006 to 31 March 2016 was partitioned into ten 1-year seasonal episodes, beginning on 1 April of one year, and ending on March of the subsequent year. Ten separate cohorts for each influenza season were constructed, comprising people with AIRDs prescribed DMARD(s). Inclusion criteria were age ⩾18 years at the start of the cohort, contributing data on 1 September of that year (start of influenza season) with ⩾3-month continuous registration at their current GP surgery prior to this date, and DMARD prescription in this period. The 3-month time lag was to allow the GP surgery time to include patients in any practice level at-risk register for influenza vaccination.

### Primary outcome

SIV administration was the primary outcome and was defined using published product and Read code lists [23]. For participants who had more than one entry for SIV administration in an influenza season, the first vaccination record was considered valid. Vaccination status of participants who contributed data to more than one influenza season returned to unvaccinated on 1 April of the subsequent year. Read codes for pandemic influenza vaccine were excluded as this study focused on temporal trends in SIV uptake.

### Secondary outcome

The secondary outcome was SIV administration by the 3rd November, that is, 9 weeks from 1 September. This would allow the individual to mount a protective immune response before mid-November when the influenza virus begins to circulate in the UK [11].

### Additional indications for SIV administration

Indications for SIV vary between countries. In the UK, SIV is indicated in those >65 years, and in those with chronic respiratory, heart, kidney, liver and neurological diseases, immunosuppression or diabetes as they are at a high risk of influenza and its complications [11]. Therefore, for this study, participants were classified as having an additional indication for vaccination if they were at least 65 years old, or had another at-risk condition that mandates SIV administration apart from DMARD prescription at the start of the influenza season. At-risk conditions were defined using Read code lists developed by Costello *et al.* [23]. These encompass chronic respiratory diseases, chronic heart diseases, chronic kidney diseases, chronic liver diseases, chronic neurological diseases, diabetes, immunosuppression and asplenia.

### Statistical analyses

The percentage and 95% CI of participants who received SIV between 1 September of one year and 31 March of the next calendar year (i.e. start and end of influenza season) was calculated. This was stratified by age (<45, 45–64, ⩾65 years), presence of other indication for SIV administration, type of AIRD (RA, SLE or SpA) and number of DMARDs prescribed (1 or >1) at annual-cohort entry. Percentage (95% CI) of vaccinations that occurred by 3 November of a year was calculated. The cumulative weekly uptake of SIV, stratified by age group and presence of other at-risk conditions was plotted using cumulative frequency curves.

Joinpoint analysis was used to determine the temporal trend in SIV uptake between 2006–07 and 2015–16 influenza seasons. This was stratified for age, AIRD type and at-risk conditions for vaccination [11]. Joinpoint uses the Bayesian information criterion to generate different numbers of joinpoints indicating points in time where trends in SIV uptake change significantly and to fit separate linear trends in each time segment. Annual percentage changes for each segment and the overall annual percentage changes (i.e. without any model fitted) were calculated.

Poisson regression with robust error variance was used to examine the univariate and multivariate associations between age, sex, AIRD type, at-risk conditions for vaccination, number of DMARDs prescribed in the preceding 12 months and receiving the SIV. A unique participant identifier was incorporated as a clustering term in the model [28]. Adjusted incidence rate ratios (aIRR) and 95% CI were calculated. This analysis was repeated using data from participants exposed to methotrexate to avoid confounding due to DMARD potency. The uptake of SIV in each geographic regions of the UK during the 2015–16 season was calculated. Poisson regression was used to examine the univariate and multivariate association between regions and SIV administration, and adjusted for age, sex, AIRD type, number of DMARDs prescribed and at-risk conditions for influenza or its complications. Data management and analysis were performed in Stata version 14 (StataCorp, College Station, TX, USA).

## Results

Data from 32 751 people with AIRDs prescribed ⩾1 DMARDs between 1 April 2006 and 31 March 2016 were included. Their mean (s.d.) age at study entry was 58.17 (14.65) years and 65.70% were female. The mean (s.d.) follow-up time was 4.37 (3.17) years. Of those included, 24 826 (75.80%) had RA, 6671(20.37%) had SpA and 1254 (3.83%) had SLE.

**Fig. 1:**
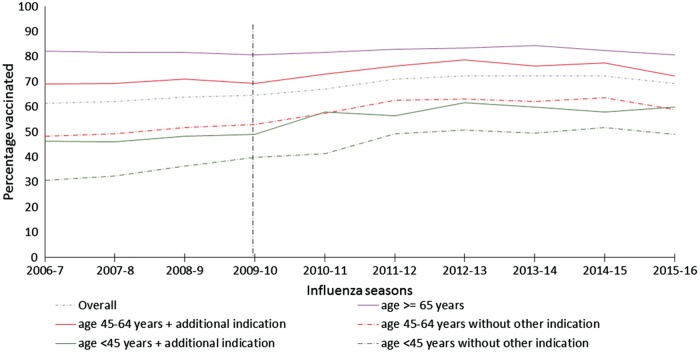
Uptake of seasonal influenza vaccine in immunosuppressed AIRD patients (2006–16) Percentage of people with autoimmune rheumatic diseases on DMARD vaccinated in each flu season from 2006 to 2016. AIRD: autoimmune rheumatic diseases.

The overall uptake of SIV increased by 2.67% (2.0–3.4%, *P* < 0.01) per year from 2006 to 2013 and remained stable between 2013 and 2015 (Table 1, Fig. 1 and supplementary Table S1, available at *Rheumatology* online). On stratified analysis, the SIV uptake did not increase in the over 65s (supplementary Table S1, available at *Rheumatology* online). However, it increased in people aged between 45 and 64 years, and in those <45 years (supplementary Table S1, available at *Rheumatology* online).

**Table 1:** Total population and percentage (95% CI) administered seasonal influenza vaccine^a^ between 2006 and 2016

Year	Overall	Age ≥65 years	Age 45–64 years, at-risk condition present	Age 45–64 years, at-risk condition absent	Age <45 years, at-risk condition present	Age <45 years, at-risk condition absent
2006–07	13 805	5658	1054	5078	192	1823
	61.52 (60.71, 62.33)	82.33 (81.31, 83.30)	69.07 (66.21, 71.79)	48.39 (47.01, 49.76)	46.35 (39.40, 53.45)	30.77 (28.70, 32.93)
2007–08	14 663	6073	1168	5355	191	1876
	62.11 (61.32, 62.89)	81.67 (80.68, 82.63)	69.43 (66.73, 72.01)	49.23 (47.89, 50.56)	46.07 (39.11, 53.19)	32.62 (30.54, 34.78)
2008–09	15 377	6468	1288	5849	209	1923
	64.04 (63.28, 64.80)	81.79 (80.83, 82.71)	71.12 (68.58, 73.53)	51.76 (50.43, 53.08)	48.33 (41.61, 55.11)	36.40 (34.28, 38.58)
2009–10	15 904	6775	1378	5572	218	1961
	64.60 (63.85, 65.34)	80.80 (79.84, 81.72)	69.38 (66.89, 71.76)	53.02 (51.70, 54.32)	49.08 (42.49, 55.71)	39.93 (37.78, 42.12)
2010–11	17 013	7170	1475	6029	236	2103
	67.10 (66.39, 67.80)	81.72 (80.80, 82.59)	73.02 (70.69, 75.22)	57.62 (56.37, 58.86)	58.05 (51.64, 64.20)	41.32 (39.23, 43.44)
2011–12	16 430	7229	1447	5643	219	1892
	71.23 (70.53, 71.92)	82.99 (82.10, 83.83)	76.36 (74.11, 78.48)	62.77 (61.50, 64.02)	56.62 (49.96, 63.05)	49.31 (47.06, 51.57)
2012–13	16 563	7409	1489	5587	224	1854
	72.30 (71.61, 72.78)	83.53 (82.67, 84.36)	78.84 (76.70, 80.85)	63.24 (61.96, 64.49)	61.61 (55.06, 67.76)	50.76 (48.48, 53.03)
2013–14	15 879	7236	1571	5140	223	1709
	72.35 (71.65, 73.04)	84.41 (83.56, 85.23)	76.38 (74.22, 78.42)	62.24 (60.90, 63.55)	60.09 (53.51, 66.33)	49.62 (47.25, 51.99)
2014–15	14 593	6784	1373	4786	178	1472
	72.41 (71.68, 73.13)	82.40 (81.47, 83.29)	77.57 (75.28, 79.70)	63.66 (62.29, 65.02)	57.87 (50.47, 64.92)	51.77 (49.21, 54.31)
2015–16	12 252	5717	1114	4049	135	1237
	69.38 (68.55, 70.19)	80.74 (79.70, 81.74)	72.26 (69.56, 74.81)	59.08 (57.55, 60.58)	60.00 (51.49, 67.94)	48.99 (46.21, 51.78)

a Vaccination period taken as from 1 September to 31 March.

**Fig. 2:**
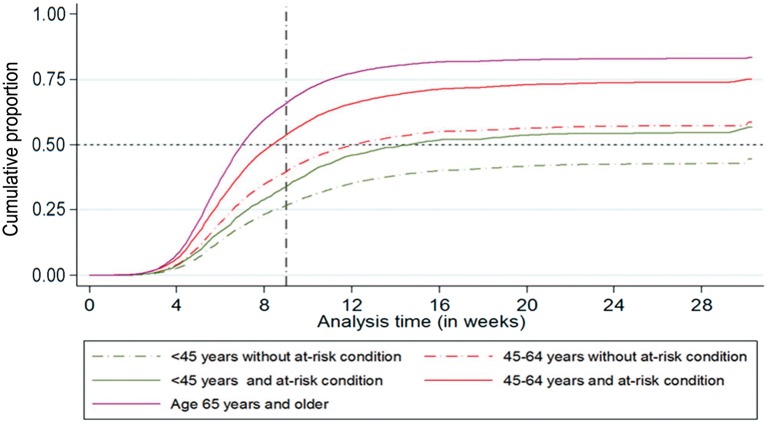
Cumulative seasonal influenza vaccine uptake between the September and March in all influenza seasons Weekly cumulative seasonal influenza vaccine uptake between 1 September and 31 March in all influenza seasons. Overall vaccine uptake was significantly higher in the over 65s (*P* < 0.001) and 45–64 year (*P* < 0.001) age group with another at-risk condition for vaccination than the 45–64 year age group without another at-risk condition for vaccination (reference group). The latter had statistically similar vaccination rates to people <45 years in age with another at-risk condition for vaccination (*P* = 0.267), while those <45 years without another at-risk condition for vaccination had significantly lower vaccination uptake than the reference group (*P* < 0.001).

**Fig. 3:**
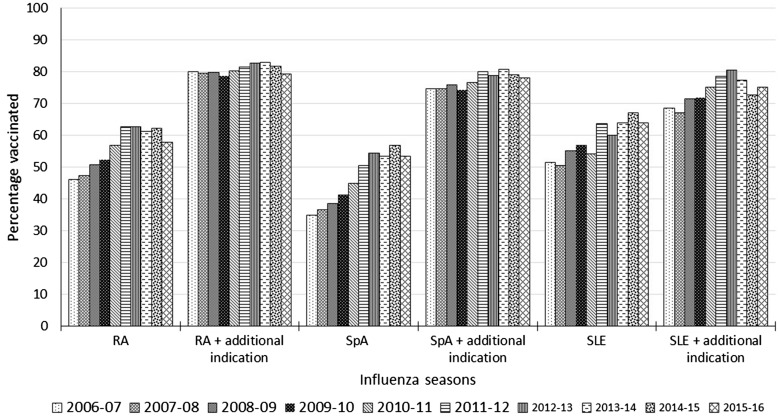
Uptake of seasonal influenza vaccine in immunosuppressed AIRD patients by disease type (2006–16) Percentage of people with AIRD on disease modifying anti-rheumatic drugs vaccinated in each flu season from 2006 to 2016 according to individual AIRD. AIRD: autoimmune rheumatic diseases.

**Fig. 4:**
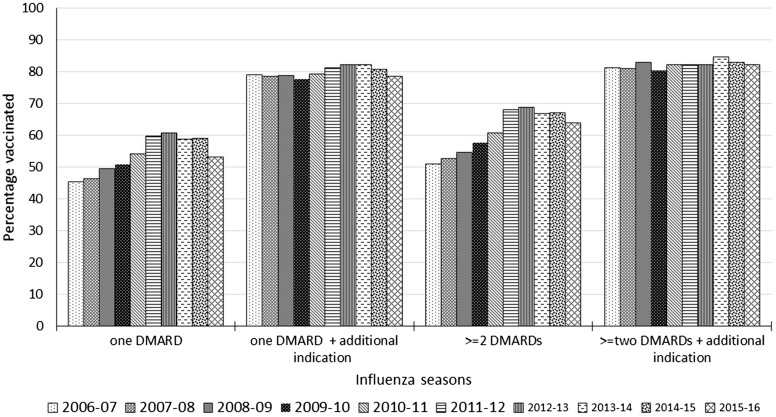
Uptake of seasonal influenza vaccine in immunosuppressed AIRD patients by number of DMARDs (2006–16) Percentage of people with AIRD on disease modifying anti-rheumatic drugs vaccinated in each flu season from 2006 to 2016 according to number of different types of DMARDs at the start of flu season. AIRD: autoimmune rheumatic diseases.

Just under half of all vaccinations (49.89%; 95% CI: 49.64, 50.14%) occurred by 3 November, that is, in time before substantial influenza activity in the UK. The proportion (95% CI) of vaccinations that happened in time was 64.93 (64.57, 65.30)% in the over 65s, 52.85 (52.01, 53.70)% and 38.89 (38.47, 39.31)% in those aged 45–64 years with and without another at-risk condition for flu vaccination, respectively, and 33.22 (31.21, 35.32)% and 26.18 (25.54, 26.84)% in those aged 45 years or younger, with and without another at-risk condition for flu vaccination, respectively (Fig. 2). The uptake of SIV was significantly higher in those with an additional indication for vaccination (Figs 3 and 4 and supplementary Tables S2 and S3, available at *Rheumatology* online).

Increasing age, female sex, presence of other at-risk conditions and prescription of more than one DMARD were independently associated with SIV administration (Table 2). After adjusting for age, sex, study year, other at-risk conditions and number of DMARDs prescribed, SLE was significantly more, and SpA significantly less likely to be associated with vaccination than RA (Table 2). These results remained unchanged when the analysis was restricted to influenza seasons with exposure to methotrexate at the annual-cohort entry, except for a lack of association between SLE and vaccination status (supplementary Table S4, available at *Rheumatology* online).

**Table 2:** Disease and demographic characteristics associated with seasonal influenza vaccine administration

Risk factors	Crude IRR (95% CI)	Adjusted IRR (95% CI)^a^
Age, years		
<45	1	1
45–64	1.35 (1.32, 1.39)	1.33 (1.29, 1.36)
≥65	1.81 (1.77, 1.86)	1.73 (1.68, 1.77)
Sex		
Male	1	1
Female	1.04 (1.03, 1.06)	1.03 (1.02, 1.05)
AIRD type		
RA	1	1
SLE	0.92 (0.88, 0.95)	1.06 (1.02, 1.10)
Seronegative spondyloarthropathy	0.81 (0.80, 0.83)	0.93 (0.92, 0.95)
Number of DMARDs		
1	1	1
≥2	1.07 (1.05, 1.09)	1.08 (1.07, 1.10)
Other influenza at-risk conditions		
Absent	1	1
Present	1.23 (1.22, 1.25)	1.12 (1.11, 1.13)

a Adjusted for study year and other variables in the table. AIRD: autoimmune rheumatic diseases; IRR: incidence rate ratio.

Geographic variation in SIV uptake was assessed in the latest flu season. In the 2015–16 influenza season, the uptake of SIV ranged between 62.58% in London and 77.74% in Scotland (supplementary Table S5, available at *Rheumatology* online). Using Poisson regression adjusted for age, sex and at-risk condition for flu vaccination, a statistically significant increased SIV uptake was observed in Northern Ireland [aIRR (95% CI): 1.16 (1.03, 1.30)] and Scotland [aIRR (95% CI): 1.15 (1.05, 1.27) compared with the South West of England (supplementary Table S5, available at *Rheumatology* online). The South West of England was used as the reference as this region had the median SIV uptake among all regions of the UK.

## Discussion

This nationwide primary-care study of SIV uptake over 10 years in people with AIRDs treated with DMARDs demonstrates an increase in uptake of SIV driven by improving uptake in people aged <65 years. However, despite this, the uptake of SIV in individuals <45 years, and in those aged between 45 and 64 years without an at-risk condition for vaccination remained significantly lower than the recommended 75% target of flu vaccination coverage for people with at-risk conditions [29–31]. Additionally, most flu vaccinations in the under 65s did not occur in time, that is, 2 weeks before the flu virus begins to circulate.

It is not possible to compare these findings with those of previous studies, since, to our knowledge, this is the first study to assess temporal trend in uptake of SIV among AIRDs and to compare uptake between different AIRDs. However, when compared with the national flu vaccination rates in England, the vaccine uptake in the under 65s in our study was higher in the year 2006–07 (national SIV uptake: 42.10% *vs* SIV uptake in study: 44.80%) and improved further (in the year 2014–15 national SIV uptake: 50.30% *vs* SIV uptake in study: 63.73%) [11]. A similar trend was observed for vaccine uptake in the over 65s, with 73.9 and 71.0% people in England receiving SIV in 2006–07 and 2015–16, respectively, compared with 82.3 and 80.7% in our study for the same years. The trend towards a reduction in vaccine uptake in the 2015–16 influenza season observed in this study was mirrored in the nationwide data [11].

The trend of increasing SIV uptake may be explained by greater public awareness, national campaigns, recommendations [9, 10] and efforts of rheumatology multi-disciplinary teams in educating people with AIRDs and their GPs about the need for SIV [14–17]. There was a substantial increase in SIV administration in the 2010–11 and 2011–12 flu seasons, especially in the <65 age group, which could be related to the pandemic flu in the year 2009–10, which increased awareness of flu vaccination.

We observed that the uptake of SIV in those without an additional reason for vaccination has improved over the 10-year period, but is still significantly lower than the uptake in those ⩾65 years or with other at-risk conditions [11]. This accords with results of previous smaller studies [13–16, 18–23, 32], and may reflect the fact that immunosuppressive DMARDs for which seasonal flu vaccination is indicated is left to individual doctors’ clinical judgement, which may result in differential vaccine access [11]. Thus, more needs to be done to improve SIV uptake in this age group. As flu vaccinations occur in primary care, there is a need to educate people with AIRDs <65 years in age, as well as their GPs, about the risks of influenza and its complications in the immunosuppressed [16, 32]. Such interventions are likely to increase SIV uptake, as people with AIRDs cite lack of awareness about increased risk of influenza, need for influenza vaccination and not being offered the vaccine as reasons for not being vaccinated [14–16, 18–20, 22, 32, 33]. Strategies to improve flu-vaccination uptake include face-to-face patient education or provision of written educational materials [odds ratio (OR) = 1.11–3.33], and financial incentives (OR = 2.22) or reminders (ORs = 2.03–3.03) directed at healthcare professionals (HCPs) [34]. Interestingly, educational outreach/feedback directed at HCPs did not improve uptake of SIV [OR (95% CI): 0.77 (0.72, 0.81)], whereas improving vaccine access with home visits or free vaccinations did improve the uptake (ORs = 1.30–1.98) [34]. Other factors such as having a lead HCP responsible for flu-vaccination in a GP surgery, robust IT systems to identify people at risk of flu and its complications, reminding HCPs about SIV and sending personal invitations and reminders, including telephone reminders to patients, associated with improved SIV uptake in a survey of 795 GP surgeries across England [35].

The ideal time for influenza vaccination in the UK is between September and early November, allowing 2 weeks for the immune response to be achieved before influenza activity becomes significant [11]. Our results, however, reveal delayed uptake of vaccination, especially in the under 65s and in those without another at-risk condition for receiving SIV. This is contrary to the findings of a claims database study from Germany in which 95% of participants who received SIV did so by November [4]. Thus, people with AIRDs and their GPs should be especially reminded about SIV in autumn months [35].

The uptake of SIV was significantly higher in people aged ⩾65 years, and was more than the target for vaccination coverage set by the Department of Health, the European Council and the WHO. This may be driven by the Quality and Outcome Framework that has provided financial incentives to GP surgeries for administering flu vaccination to people aged ⩾65 years since the 1999–2000 influenza season. It resulted in a substantial increase in vaccination rates within 1 year [36]. Other factors such as a high burden of co-morbidities and availability of time may also have contributed. A previous study from Germany also demonstrated increasing SIV uptake with increasing age [4].

Our study revealed variations in the uptake of influenza vaccination according to AIRD type, with lower uptake in SpA. This was present on sensitivity analysis, restricting to influenza seasons in which a MTX prescription was issued. This suggests that greater effort should be employed to improve vaccination rates in people with SpA, who are predominantly male and less likely to seek preventive measures.

There are several caveats to our study. Firstly, some flu vaccinations may have been administered in a hospital or at the work-place, for example, vaccinations in health care professionals. This is unlikely to have a significant impact as SIV is administered predominantly in primary care in the UK, and Read codes that indicate SIV administration outside the GP surgery, that is, in a hospital, place of work or privately, were included in the code list. Moreover, we excluded small vessel vasculitis because of the rarity of its recording in the CPRD [37]. Similarly, given their extremely rare use in modern rheumatology practice, we excluded penicillamine and gold prescriptions. Finally, the data on prescription of biologic agents is not recorded in the CPRD, and for this reason we cannot compare SIV uptake in those prescribed biologic and conventional DMARDs. However, a previous German study did not report higher SIV uptake in people on biologic DMARDs compared with conventional DMARDs [4].

There are several strengths of this study, which include a large nationally representative sample size, use of a combination of diagnostic Read codes and DMARD prescriptions to identify immunosuppressed people with AIRDs, inclusion of a broad spectrum of AIRDs and a longitudinal observation period of 10 years. Use of primary care prescription and consultation data minimizes the risk of recall bias associated with questionnaire surveys.

In summary, this study demonstrates improving but still low uptake of SIV in people with AIRDs <65 years. Similarly, the majority of people in this age group are vaccinated after the seasonal flu virus begins to circulate. Thus, rheumatologists and GPs should particularly educate younger people with AIRDs about the need for timely annual seasonal flu vaccination.

*Funding*: This work was supported by the Arthritis Research UK [grant number 21297]; and the National Institute of Health Research [NIHR-RP-2014-04-026].

*Disclosure statement*: C.M. is funded by the National Institute for Health Research (NIHR) Collaborations for Leadership in Applied Health Research and Care West Midlands, the NIHR School for Primary Care Research and a NIHR Research Professorship in General Practice (NIHR-RP-2014-04-026); the views expressed in this manuscript are those of the author(s) and not necessarily those of the NHS, the NIHR or the Department of Health. J.S.N.-V.-T. is on secondment to the Department of Health, England; the views expressed in this manuscript do not represent the official position of the Department of Health. W.Z. has received honorariums from AstraZeneca and Grunenthal and speaker fees from Biobarica and Hisun unrelated to this work. A.A. has received departmental research grants from AstraZeneca and Oxford Immunotech and speaker bureau fees from Menarini unrelated to this work. M.D. has attended ad hoc advisory boards on osteoarthritis or gout for AstraZeneca, Grunenthal, Mallinckrodt and Roche and is an Investigator in an AstraZeneca funded, investigator-led, non-drug study (the ‘Sons of Gout’ study). P.M. is an employee of Medicines and Healthcare Products Regulatory Agency (MHRA) but MHRA did not play any role in the conduct or reporting of this study. All other authors have declared no conflicts of interest.

## Supplementary Material

Supplementary Data
