# Supplementary material for: Predictors and temporal trend of flu vaccination in auto-immune rheumatic diseases in the UK: a nationwide prospective cohort study
Source: Rheumatology (Oxford). 2018 Jun 12;57(10):1726–34. doi: 10.1093/rheumatology/key156 (PMC6152422; doi:10.1093/rheumatology/key156)
Supplement: Supplementary Data [file key156_suppl_data.docx]

**Supplementary Data**

**Supplementary Table S1: Annual percentage change in seasonal influenza vaccine uptake 2006-2016**

| Age group | At risk conditions | Significant joinpoints | Start | End | APC | Lower 95% CI | Upper 95% CI | P-value |
| --- | --- | --- | --- | --- | --- | --- | --- | --- |
| Overall | | 2013-14 | 2006 | 2013 | 2.7^b^ | 2.0 | 3.4 | <0.01 |
|  |  |  | 2013 | 2015 | -2.7 | -7.7 | 2.5 | 0.20 |
| ≥65 years^a^ | | none | 2006 | 2015 | 0.1 | -0.2 | 0.5 | 0.40 |
| 45-64 years | + | 2011-12 | 2006 | 2011 | 6.1^b^ | 3.7 | 8.6 | <0.01 |
|  |  |  | 2011 | 2015 | -0.3 | -3.2 | 2.7 | 0.80 |
| 45-64 years | - | none | 2006 | 2015 | 1.3^b^ | 0.1 | 2.6 | <0.01 |
| <45 years | + | 2012-13 | 2006 | 2012 | 8.8^b^ | 6.2 | 11.4 | <0.01 |
|  |  |  | 2012 | 2015 | -0.2 | -4.6 | 4.5 | 0.90 |
| <45 years | - | 2012-13 | 2006 | 2012 | 5.4^b^ | 2.4 | 8.5 | <0.01 |
|  |  |  | 2012 | 2015 | -1.5 | -8.7 | 6.3 | 0.60 |

^a^People aged 65 years or greater in the UK are offered influenza vaccination regardless of comorbidities. ^b^Indicates that the annual Percent change (APC) is significantly different from zero at the alpha=0.05 level

**Supplementary Table S2: Administration of seasonal influenza vaccine according to autoimmune rheumatic disease type**

| Year | RA  additional indication ­–ve | SLE  additional indication -ve | SpA  additional indication –ve | RA  additional indication  +ve | SLE  additional indication  +ve | SpA additional indication  +ve |
| --- | --- | --- | --- | --- | --- | --- |
| 2006/7 | 4,998  46.08(44.70-47.46) | 301  51.50 (45.84-57.11) | 1,602  34.96 (32.66-37.33) | 6,132  80.06 (79.04-81.04) | 152  68.42 (60.59-75.33) | 620  74.52 (70.93-77.80) |
| 2007/8 | 5,176  47.39(46.03-48.75) | 317  50.47(44.98-55.96) | 1,738  36.54(34.30-38.83) | 6,568  79.57(78.57-80.53) | 164  67.07(59.50-73.85) | 700  74.71(71.36-77.80) |
| 2008/9 | 5,226  50.63 (49.27-51.99) | 326  55.21(49.77-60.54) | 1,860  38.44(36.25-40.68) | 6,987  79.78 (78.82-80.70) | 175  71.43 (64.28-77.65) | 803  75.72 (72.63-78.56) |
| 2009/10 | 5,228  52.31 (50.96-53.67) | 331  56.80 (51.39-62.04) | 1,974  41.24 (39.08-43.42) | 7,299  78.71 (77.75-79.63) | 181  71.82 (64.82-77.91) | 891  74.30 (71.32-77.06) |
| 2010/11 | 5,578  56.72 (55.42-58.02) | 374  54.01 (48.93-59.01) | 2,180  44.82 (42.74-46.91) | 7,648  80.19 (79.28-81.07) | 200  75.00 (68.52-80.53) | 1,033  76.48 (73.79-78.97) |
| 2011/12 | 5,126  62.76 (61.43-64.07) | 319  63.64 (58.20-68.74) | 2,090  50.48 (48.33-52.62) | 7,617  81.52 (80.63-82.37) | 201  78.61 (72.38-83.75) | 1,077  79.94 (77.44-82.23) |
| 2012/13 | 4,965  62.69 (61.34-64.03) | 310  60 (54.43-65.32) | 2,166  54.25 (52.14-56.34) | 7,790  82.76 (81.90-83.58) | 220  80.45 (74.67-85.18) | 1,112  78.87 (76.37-81.17) |
| 2013/14 | 4,569  61.28 (59.86-62.69) | 271  63.84 (57.93-69.35) | 2,009  53.46 (51.27-55.63) | 7,678  82.82 (81.96-83.65) | 215  77.21 (71.11-82.34) | 1,137  80.65 (78.25-82.85) |
| 2014/15 | 4,168  62.24 (60.75-63.70) | 257  66.93 (60.93-72.42) | 1,833  56.90 (54.62-59.15) | 7,016  81.67 (80.75-82.56) | 197  72.59 (65.93-78.38) | 1,122  78.88 (76.39-81.17) |
| 2015/16 | 3,463  57.75 (56.10-59.39) | 229  63.76 (57.31-69.74) | 1,594  53.45 (50.99-55.89) | 5,857  79.24 (78.18-80.26) | 161  75.16 (67.88-81.24) | 948  78.06 (75.31-80.58) |

**Supplementary Table S3: Administration of seasonal influenza vaccine stratified by number of prescribed DMARDs**

| Year | 1 DMARD, no additional indications | >1 DMARD, no  additional indications | 1 DMARD and additional indications | >1 DMARD and  additional indications |
| --- | --- | --- | --- | --- |
| 2006/7 | 5,985  42.64(41.39-43.90) | 916  50.87(47.63-54.10) | 6,156  79.06(78.03-80.06) | 748  81.28(78.32-83.92) |
| 2007/8 | 6,255  43.73(42.50-44.96) | 976  52.56(49.42-55.68) | 6,560  78.55(77.54-79.53) | 872  80.96(78.22-83.44) |
| 2008/9 | 6,342  46.61 (45.38-47.84) | 1,070  54.67 (51.67-57.64) | 7,006  78.69 (77.71-79.63) | 959  82.79 (80.27-85.06) |
| 2009/10 | 6,401  48.18 (46.96-49.41) | 1,132  57.69 (54.78-60.54) | 7,300  77.75 (76.78-78.69) | 1,071  80.39 (77.90-82.66) |
| 2010/11 | 6,853  52.05 (50.87-53.23) | 1,279  60.67 (57.96-63.32) | 7,725  79.28 (78.36-80.16) | 1,156  82.09 (79.77-84.20) |
| 2011/12 | 6,379  57.81 (56.60-59.02) | 1,156  68.08 (65.33-70.71) | 7,745  81.11 (80.22-81.97) | 1,150  82.26 (79.94-84.36) |
| 2012/13 | 6,336  58.60 (57.38-59.81) | 1,105  68.87 (66.07-71.53) | 7,960  82.24 (81.38-83.06) | 1,162  82.19 (79.88-84.28) |
| 2013/14 | 5,826  57.74 (56.47-59.00) | 1,023  66.76 (63.82-69.59) | 7,878  82.08 (81.21-82.91) | 1,152  84.72 (82.53-86.69) |
| 2014/15 | 5,359  59.82 (58.50-61.13) | 899  67.07 (63.93-70.07) | 7,258  80.79 (79.87-81.68) | 1,077  83.01 (80.64-85.14) |
| 2015/16 | 4,554  55.56 (54.11-56.99) | 732  63.93 (60.38-67.34) | 6,092  78.51 (77.46-79.53) | 874  82.27 (79.59-84.66) |

**Supplementary Table S4: Disease and demographic characteristics associated with seasonal influenza vaccine administration in patients using methotrexate**

| Characteristic | Crude IRR (95% CI) | Adjusted IRR (95% CI)^a^ |
| --- | --- | --- |
| **Age, years** |  |  |
| <45 | 1 | 1 |
| 45-64 | 1.26 (1.23-1.30) | 1.24 (1.21-1.28) |
| ≥65 | 1.59 (1.54-1.63) | 1.53 (1.49-1.57) |
| **Sex** |  |  |
| Male | 1 | 1 |
| Female | 1.03 (1.01-1.04) | 1.03 (1.01-1.04) |
| **AIRD type** |  |  |
| Rheumatoid arthritis | 1 | 1 |
| Systemic lupus erythematosus | 0.92 (0.86-0.98) | 1.02 (0.95-1.08) |
| Seronegative spondyloarthropathy | 0.87 (0.85-0.87) | 0.96 (0.94-0.98) |
| **Number of DMARDs** |  |  |
| 1 | 1 | 1 |
| >1 | 1.02 (1.00-1.03) | 1.03 (1.02-1.05) |
| **Other influenza at-risk condition** |  |  |
| Absent | 1 | 1 |
| Present | 1.18 (1.16-1.19) | 1.09 (1.08-1.10) |

^a^Adjusted for study year and other variables in the table. AIRD: autoimmune rheumatic diseases; IRR: Incidence rate ratios.

**Supplementary Table S5: Vaccinated with seasonal influenza vaccine in the 2015/16 influenza season stratified by geographic region**

| Region | Percentage (95% CI) | Crude IRR (95% CI) | Adjusted IRR (95% CI)^a^ |
| --- | --- | --- | --- |
| South West^b^ | 68.77 (65.42-71.94) | 1 | 1 |
| North West England | 69.27 (66.45-71.96) | 1.01 (0.90-1.13) | 1.00 (0.89-1.11) |
| Yorkshire and Humber | 72.64 (63.35-80.31) | 1.06 (0.83-1.34) | 1.04 (0.82-1.32) |
| West Midlands | 64.19 (60.95-67.30) | 0.93 (0.83-1.05) | 0.95 (0.84-1.07) |
| East Midlands | - | - | - |
| East England | 65.28 (60.80-69.51) | 0.95 (0.82-1.09) | 0.95 (0.83-1.00) |
| South Central | 66.49 (64.10-68.81) | 0.97 (0.87-1.07) | 0.99 (0.89-1.10) |
| London | 62.58 (59.20-65.84) | 0.91 (0.81-1.03) | 0.92 (0.82-1.04) |
| South East Coast | 66.02 (63.69-68.27) | 0.96 (0.87-1.06) | 0.96 (0.87-1.07) |
| Northern Ireland | 76.53 (73.53-79.29) | 1.11 (0.99-1.25) | 1.16 (1.03-1.30) |
| Scotland | 77.74 (75.91-79.46) | 1.13 (1.03-1.25) | 1.15 (1.05-1.27) |
| Wales | 68.59 (66.49-70.62) | 1.00 (0.90-1.10) | 1.01 (0.91-1.11) |
| North East England | 70.97 (60.91-79.32) | 1.03 (0.78-1.33) | 1.00 (0.77-1.29) |

**^a^**Adjusted for age at start of vaccination period, sex and presence of one or more risk condition for influenza vaccination at start of vaccination period. ^b^Region with a median vaccinated population. No data from the East Midlands region. Statistical significance determined at p<0.05.
